# The Neuroscience Behind Writing: Handwriting vs. Typing—Who Wins the Battle?

**DOI:** 10.3390/life15030345

**Published:** 2025-02-22

**Authors:** Giuseppe Marano, Georgios D. Kotzalidis, Francesco Maria Lisci, Maria Benedetta Anesini, Sara Rossi, Sara Barbonetti, Andrea Cangini, Alice Ronsisvalle, Laura Artuso, Cecilia Falsini, Romina Caso, Giuseppe Mandracchia, Caterina Brisi, Gianandrea Traversi, Osvaldo Mazza, Roberto Pola, Gabriele Sani, Eugenio Maria Mercuri, Eleonora Gaetani, Marianna Mazza

**Affiliations:** 1 Unit of Psychiatry, Fondazione Policlinico Universitario AgostinoGemelli IRCCS, 00168 Rome, Italyfmlisci@gmail.com (F.M.L.); mbenedetta@hotmail.it (M.B.A.); sara.barbonetti@gmail.com (S.B.);; 2 Department of Neurosciences, Università Cattolica del Sacro Cuore, 00168 Rome, Italy; 3 Accademia di Psicologia e Espressione Della Scrittura, 00168 Rome, Italy; aliceronsisvalle99@gmail.com (A.R.);; 4 Fondazione Luigi Einaudi, 00193 Rome, Italy; segretariogenerale@fondazioneluigieinaudi.it; 5 Osservatorio Carta, Penna & Digitale, 00193 Rome, Italy; 6 Unit of Medical Genetics, Department of Laboratory Medicine, Ospedale Isola Tiberina-Gemelli Isola, 00186 Rome, Italy; 7 Spine Surgery Department, Bambino Gesù Children’s Hospital IRCCS, 00168 Rome, Italy; osvaldo.mazza1973@hotmail.it; 8 Section of Internal Medicine and Thromboembolic Diseases, Department of Internal Medicine, Fondazione Policlinico Universitario Agostino Gemelli IRCCS, Università Cattolica del Sacro Cuore, 00168 Rome, Italy; 9 Department Women Children and Public Health, Università Cattolica del Sacro Cuore, 00168 Rome, Italy; eugeniomaria.mercuri@unicatt.it; 10 Department of Translational Medicine and Surgery, Fondazione Policlinico Universitario AgostinoGemelli IRCCS Università Cattolica del Sacro Cuore, 00168 Rome, Italy; 11 Unit of Internal Medicine, Cristo Re Hospital, 00167 Rome, Italy

**Keywords:** handwriting, typing, magnetic resonance imaging, functional, electroencephalography, cognition

## Abstract

Background: The advent of digital technology has significantly altered ways of writing. While typing has become the dominant mode of written communication, handwriting remains a fundamental human skill, and its profound impact on cognitive processes continues to be a topic of intense scientific scrutiny. Methods: This paper investigates the neural mechanisms underlying handwriting and typing, exploring the distinct cognitive and neurological benefits associated with each. By synthesizing findings from neuroimaging studies, we explore how handwriting and typing differentially activate brain regions associated with motor control, sensory perception, and higher-order cognitive functions. Results: Handwriting activates a broader network of brain regions involved in motor, sensory, and cognitive processing. Typing engages fewer neural circuits, resulting in more passive cognitive engagement. Despite the advantages of typing in terms of speed and convenience, handwriting remains an important tool for learning and memory retention, particularly in educational contexts. Conclusions: This review contributes to the ongoing debate about the role of technology in education and cognitive development. By understanding the neural differences between handwriting and typing, we can gain insights into optimal learning strategies and potential cognitive advantages, in order to optimize educational, cognitive, and psychological methodologies.

## 1. Introduction

Handwriting is a complex neurophysiological process integrating motor, cognitive, and emotional components. Beyond its functional role in communication, it carries cultural and psychological significance [1,2]. Writing systems vary significantly across cultures, and an important distinction exists between alphabet-based and hieroglyphic writing systems. Alphabet-based writing systems, such as English or Italian, rely on a relatively small set of symbols (letters) that combine systematically to form words. In contrast, hieroglyphic and logographic systems (e.g., Chinese, ancient Egyptian) require the retrieval of complex, visually distinct symbols that each represent a concept or word. Neuroimaging studies indicate that writing in logographic systems engages different brain regions compared with alphabetic writing. Specifically, writing Chinese characters or hieroglyphics involves increased activation of the right hemisphere, particularly in visuospatial processing areas, due to the intricate and non-linear nature of the script. This is in contrast with alphabetic systems, which predominantly engage the left hemisphere, particularly in phonological processing areas. The cognitive demands of writing hieroglyphic characters may lead to enhanced visuospatial memory and motor coordination compared with alphabet-based writing, highlighting significant differences in neural processing between these two systems [3].

Characteristics of handwriting such as size, pressure, slant, and spacing can reveal insights into a person’s traits. Handwriting analysis, also known as graphology, is a method of identifying the traits of an individual through his/her handwriting [4]. Graphologists study these features to analyse personality, emotional tendencies, and even stress levels. While the scientific validity of graphology remains debated, handwriting undeniably showcases nuances of the writer’s mental and physical condition at the time of writing.

Handwriting is an acquired skill of language production, through which external stimuli (visual or auditory) are translated into a fluid and coordinated trace on a sheet of paper. It is primarily a process, and then a product: handwriting involves the coordination of linguistic, motor, and visuospatial processes, a set of operations that allow abstract orthographic representations to be mapped into graphomotor patterns distributed over a two-dimensional space [5]. Linguistic processes involve activation of orthographic representation via words, the conversion of phonemes (the sounds corresponding to each letter) into graphemes (written symbols), and the maintenance of abstract graphemic representations in the graphemic buffer, a memory store where orthographic representations of words can remain active for a short time. Meanwhile, motor and visuospatial processes encompass motor planning of hand movements, their transformation into motor commands, fine motor control of gesture, visuomotor integration (i.e., the coordination between visual perception and hand movements), proprioception (the perception of the hand’s position and movement independent of sight), and sensory awareness of the fingers. Handwriting necessitates the execution of a gesture that, like any motor act, requires the mobilization of neural networks. The extreme precision of the graphic gesture distinguishes it from other movements; it represents a highly precise act of fine motor skill that humans acquire through extensive learning and maturation over time [6]. Consequently, handwriting is an acquired skill. With experience, the child gradually improves in both linguistic aspects (phonemic–graphemic conversion) and motor aspects (hand–eye coordination), transitioning from a voluntarily and retrospectively controlled movement to an anticipatory controlled movement. In adolescents and adults, as the graphic gesture becomes increasingly automated and spontaneous, error detection occurs concurrently with the movement [6].

Culturally, handwriting has evolved alongside technology, shifting from a daily necessity to an art form in some contexts. In historical records, it serves as a window into the past, preserving traditions, emotions, and the essence of an era. Modern technologies like keyboards and digital notetaking have reduced reliance on handwriting, but its significance persists, especially in personal and creative domains.

From an evolutionary perspective, handwriting evolved from fine motor control needed for tool use and from early forms of symbolic representation such as cave drawings. These activities required precise hand movements and were closely tied to the development of human manual dexterity. Typing is a relatively recent skill in evolutionary terms, emerging with the invention of typewriters and computers. It builds on gross motor skills (finger tapping) rather than fine motor control, and thus it relies less on the evolutionary development of manual dexterity. Writing involves producing unique shapes that require individual attention and creativity, reflecting the gradual development of symbolic communication over millennia, while typing relies on pressing predefined keys, abstracting the act of communication from the shape of symbols, which reduces the cognitive load of creating unique forms [7].

The neural mechanisms underlying handwriting are intricately linked to perceptual, motor, and cognitive functions, providing a rich substrate for the expression of thoughts and emotions. Laganaro et al. [8] investigated the use of graph signals, handwriting, and voice signals for the classification of depressive disorder patients, demonstrating the potential of this multimodal approach for improved diagnostic accuracy. In subsequent work, Laganaro et al. [9] further expanded on these findings by developing machine learning models based on graphophonological features specifically for the detection of depressive disorder, highlighting the promising potential of this approach for clinical applications. Recent studies have explored the potential of handwriting analysis as a non-invasive biomarker for neurological disorders such as Parkinson’s disease (PD). Mancini et al. [10] developed machine learning models based on graph signals for PD screening and telemonitoring. Similarly, Fratello et al. [11] investigated the use of classification algorithms to screen for PD using graph and handwriting features, demonstrating promising results in differentiating between PD patients and healthy controls. 

Recent neuroimaging studies including functional magnetic resonance imaging (fMRI), electroencephalography (EEG), and positron emission tomography (PET) have provided insights into the neural mechanisms that underlie these two forms of written expression. To ensure clarity in the comparison of handwriting and typing, it is essential to define these terms precisely. “Handwriting” denotes the manual production of written symbols through fine motor coordination, traditionally performed with a pen or pencil on paper. However, in modern contexts, handwriting also includes writing with a digital stylus on touchscreen devices, which may engage additional neural mechanisms due to variations in sensory feedback and motor execution. In contrast, “typing” involves the selection of pre-formed characters via a keyboard, relying on distinct motor processes including procedural memory and repetitive finger tapping, rather than continuous manual tracing of letterforms. These fundamental differences in motor execution necessitate careful consideration when examining brain activation patterns in neuroimaging studies, revealing significant differences in brain activation patterns when individuals engage in handwriting versus typing, suggesting that the two modes of writing may contribute differently to cognitive processes such as memory retention, learning, and language processing [12,13]. 

Traditional handwriting with pen and paper engages fine motor skills and proprioceptive feedback from the physical resistance of the writing surface. Conversely, digital handwriting performed with a stylus on a touchscreen modifies this interaction by reducing tactile resistance while introducing distinct haptic feedback mechanisms. This distinction is particularly relevant as digital writing technologies become increasingly prevalent in educational and professional settings. As these tools reshape the learning environment, it is crucial to consider their implications for educational practices, especially in relation to the neural mechanisms underlying handwriting. Emerging technologies such as touchscreen devices and digital pens are reshaping sensory experiences by introducing new forms of interaction that differ from traditional handwriting or typing. These technologies alter sensory feedback, engagement, and perception in various ways. Traditional touchscreens offer limited tactile feedback, which can affect the user’s sensory experience. To address this, researchers have developed systems that provide back-of-device force feedback to enhance the accuracy of touchscreen interaction. For example, a study introduced a back-of-device force feedback system for smartphones, utilizing actuated pins to improve precision selection of small or hidden objects [14]. A study found that once individuals are accustomed to it, handwriting with a digital pen and tablet can increase the ability to learn compared with keyboard typing [15]. Digital handwriting on touchscreens offers a compelling alternative that leverages the strengths of both traditional handwriting and typing, providing cognitive benefits alongside modern convenience. These pens offer enhanced haptic feedback through pressure sensitivity, allowing users to feel a closer simulation of real pen-on-paper writing. A study on a pen-type multi-mode haptic interface for touchscreen interaction demonstrated that integrating actuators can provide vibrotactile feedback and precise force feedback, enhancing the realism of digital handwriting tasks [16]. The sensory feedback they offer can be less tactile than traditional pen-on-paper writing, though advances in haptic feedback are gradually closing this gap. The ability of digital pens to offer a similar experience to traditional handwriting while providing the advantages of digital flexibility is particularly promising for learning and memory retention.

### 1.1. Handwriting Versus Typing

Handwriting and typing engage different cognitive and neural mechanisms, with implications from both evolutionary and neuroscientific perspectives. From a neuroscientific perspective, studies have shown that handwriting engages the sensorimotor cortex, visual areas, and language centres (e.g., Broca’s area) more extensively than typing. This is because forming letters requires integrating visual and tactile feedback as well as motor planning [14]. Typing predominantly activates motor regions associated with repetitive finger movements and visual processing, with less direct engagement of areas associated with memory and language. Handwriting can also be broadly categorized into cursive (joined-up writing) and block letters (non-joined writing). From a neurocognitive perspective, cursive writing is a more fluid and continuous motor activity, engaging different neural circuits than block letters. Research suggests that cursive writing involves greater activation of the motor cortex and cerebellum, as it requires fine motor coordination and smooth transitions between letters. This fluidity enhances memory retention and cognitive engagement, as the writer must anticipate the next letter in a continuous motion. Conversely, block-letter handwriting, which involves lifting the pen after each letter, may engage discrete motor planning processes and different visual-spatial coordination patterns. A study using high-density EEG demonstrated that cursive writing recruits broader neural networks compared with block-letter writing, which may have implications for education and cognitive development [17].

Neuroimaging studies indicate that the distinction between handwriting and typing is not always clear-cut and depends on multiple factors. For instance, in an fMRI study on 12 right-handed adults, Beeson et al. [18] demonstrated a distinct functional specialization of the left superior parietal cortex for handwriting. In contrast, Roux et al. [19] studied 24 participants via fMRI and found similar activation in the same area during typing, suggesting that some aspects of orthographic encoding are shared between both modalities. Furthermore, technological familiarity plays a crucial role. Nakamura et al. [20] examined nine Japanese speakers using fMRI and found increased activation in the left temporo-occipital cortex in individuals using ideograms compared with those employing phonetic kana script, indicating that the type of writing system influenced the brain activity. This contrasted with findings from Segal and Petrides [21], who studied nine bilingual English–French speakers and observed that typing activated a similar motor network regardless of the language used, suggesting a lower influence of linguistic specificity in typing compared with handwriting. These discrepancies imply that the cognitive impact of handwriting and typing may vary not only based on motor execution but also according to individual experience, native language, and familiarity with the technology used.

Writing by hand strengthens memory and learning through the “encoding effect”, where the effort of forming letters improves retention and comprehension. Using fMRI, Longcamp et al. [22] found that handwriting enhanced the encoding of new characters due to greater engagement of the left fusiform gyrus and the superior parietal lobule, areas associated with visual word recognition and spatial processing. In contrast, Mueller and Oppenheimer [7], using behavioral analysis rather than neuroimaging, demonstrated that students who took handwritten notes retained conceptual information better than those who typed, even when typing speed was controlled. However, the extent of this memory advantage varied depending on the complexity of the writing task. Palmis et al. [23] found that writing irregular words activated motor-related regions, such as the superior frontal gyrus and cerebellum, more than regular words, indicating that greater cognitive effort during handwriting might be a key driver of its memory benefits. This finding is consistent with the work of Planton et al. [5], who demonstrated that writing activates a larger visuomotor network compared with typing, suggesting that multisensory integration plays a crucial role in memory encoding. Together, these studies suggest that handwriting may confer memory advantages not only due to deeper encoding but also due to greater engagement of neural circuits involved in motor control, visuospatial integration, and semantic processing. This is particularly evident in children learning to write, as handwriting supports the development of neural pathways associated with literacy. Furthermore, while efficient for recording large amounts of information, typing does not engage the brain’s memory systems so deeply. Research has shown that typists tend to transcribe verbatim, which results in shallower processing of information [15]. Furthermore, it is essential to adopt a more nuanced perspective on the cognitive implications of handwriting. Although handwriting is frequently associated with improved memory retention, this effect may to some extent stem from the cognitive engagement required for the aesthetic and graphical aspects of letter formation rather than the semantic processing of content. The attentional focus on visual and stylistic elements such as letter shape and spatial connectivity, may impose an additional cognitive load that could inadvertently divert cognitive resources away from the comprehension and encoding of core information content, potentially diminishing rather than enhancing retention. More importantly, the cognitive benefits attributed to handwriting may not be a direct consequence of the act itself but rather a function of the graphical representation of information. Handwriting often extends beyond mere transcription, incorporating implicit or explicit cognitive structuring through the creation of mind maps, diagrams, and conceptual frameworks, thereby engaging multiple brain regions concurrently. This integrative encoding process facilitates the transformation of isolated data points into structured and interconnected networks of knowledge. It is conceivable that this distributed neural activation, rather than the motor execution of handwriting alone, underlies its positive effects on learning and long-term memory consolidation. Research suggests that handwriting may offer specific advantages over typing especially when it comes to memory retention and comprehension. Handwriting has been shown to enhance memory retention compared with typing. This is largely due to the fact that writing by hand is a more cognitively demanding process, requiring the brain to engage in deep encoding of the information. When typing, individuals tend to transcribe information passively, often without engaging deeply with the material. Handwriting slows down the notetaking process, prompting the subject to actively paraphrase and process the material rather than merely transcribe it. In addition, positive mood during learning is significantly higher during handwriting than during typing; so, the advantage of handwriting over typing might also be caused by a more positive mood during learning [7]. For children and beginners, handwriting is often found to be more beneficial than typing. This is because the motor skills involved in writing by hand can improve cognitive abilities like letter recognition and spelling. Handwriting promotes better learning of new words and concepts, as it combines cognitive, sensory, and motor elements in a way that typing does not. Research found that children who practiced writing by hand had better reading fluency and comprehension than those who practiced typing, particularly when learning letters and sounds [24]. Studies using brain imaging have shown that handwriting engages more areas of the brain associated with creativity and critical thinking, which may be because handwriting permits a more flexible and personalized approach to notetaking and problem-solving. While typing is certainly faster and more efficient in many contexts, handwriting can offer distinct cognitive benefits, particularly for tasks involving memory retention, comprehension, critical thinking, and creativity. It engages different parts of the brain, requiring more active processing and, in some cases, promoting deeper learning.

The slower pace of handwriting fosters deeper thought and creativity, as the brain has more time to process and synthesize ideas. It is also true that typing speed may favour brainstorming and fast generation of ideas but can lead to less reflective processing [16].

Handwriting is a dynamic motor process that engages a network of brain regions primarily associated with motor control, visuospatial coordination, and language processing. Studies employing fMRI and transcranial magnetic stimulation (TMS) have identified in the primary motor cortex, the premotor cortex, the supplementary motor area (SMA), and the cerebellum as the core areas controlling the execution and refinement of handwriting movements. In addition, the parietal lobe facilitates visuospatial integration, while Broca’s area and other language-related cortical regions contribute to orthographic encoding and graphemic representation during handwriting tasks [14,16]. In contrast, typing is characterized by selecting and arranging pre-formed symbols rather than producing them manually, relying on a distinct combination of motor and cognitive functions. This modality involves greater engagement of the prefrontal cortex for decision-making and attention, as well as posterior parietal regions for spatial organization. While typing requires less fine motor control, its dependence on working memory and executive functions is pronounced, reflecting the task’s reliance on higher-order cognitive processes [17]. Despite these differences, handwriting and typing both depend on shared neural pathways for visual and orthographic processing. The ventral occipitotemporal cortex, including the so-called “visual word form area” (VWFA), integrates the recognition and interpretation of written symbols. This area, in conjunction with temporal and parietal regions, supports the neural encoding of written language across modalities [18].

An essential aspect to consider is how prolonged use of handwriting versus typing influences brain adaptation, motor skills, and language development. In modern society where typing has become increasingly dominant, it is crucial to investigate how this mode of writing shapes cognitive development and neuroplasticity. The growth and organization of the brain are influenced by both intrinsic genetic factors and external sensory experiences. According to the study by Cisneros-Franco et al. [25], the influence of sensory input on brain structure and function is strongly dependent on developmental stages, as neural plasticity fluctuates throughout life. In early development, before many essential neural circuits have been fully established, the brain exhibits heightened plasticity, enabling rapid adaptation and learning. In contrast, the adult brain is more structurally stable, making the acquisition of new skills more challenging. This shift from a highly flexible state to a more fixed one is beneficial as it supports the progressive consolidation and retention of increasingly complex perceptual, motor, and cognitive functions.

Some researchers have hypothesized that increased reliance on keyboard-based writing may reduce activation in neural circuits involved in learning and memory consolidation. Conversely, handwriting, which requires greater motor and cognitive engagement, may confer long-term benefits for brain function preservation. Exploring the long-term effects of these activities on brain structure and function could further strengthen our understanding of their neurocognitive implications. Neuroimaging techniques have revealed significant differences in brain activation patterns between individuals who engage in handwriting and those who primarily type, suggesting that the mode of writing may shape neural architecture over time. Understanding these changes could have important applications in education, cognitive rehabilitation, and the development of strategies to optimize learning outcomes.

The transition from handwriting to typing in early education may have significant implications for children’s long-term motor skills and cognitive development. Handwriting engages fine motor skills and cognitive processes that are crucial for reading and writing proficiency. Research indicates that handwriting and reading share neural pathways, and the cognitive processes involved in handwriting, such as letter formation and letter–sound relationships, are also involved in reading acquisition. Additionally, the physical act of writing helps children develop visual and spatial awareness, fine motor skills, and hand-eye coordination, which are important for the reading process. In contrast, typing primarily involves repetitive movements and may not provide the same level of fine motor skill development. If children are not exposed to handwriting early on, they may not develop the precise hand movements needed for later tasks such as drawing, cutting, or other manual activities. The lack of varied fine motor tasks in their early years could lead to delayed development of dexterity and hand strength, which are critical for daily life and future academic or professional tasks. Handwriting builds muscle memory through repetitive, intricate finger movements. Without practice, children may miss out on developing the fine motor control necessary for activities that require precision, such as playing musical instruments, drawing, or even participating in physical activities like sports. Typing requires less varied motor movement than handwriting, which involves shaping letters and adjusting pen pressure, and this could result in different patterns of neural development associated with motor control and coordination. The shift toward typing in early education may prioritize speed and efficiency over deep learning and cognitive engagement.

Understanding the interplay of these cerebral structures is critical not only for elucidating the neural basis of written communication but also for addressing cognitive and motor deficits that impair writing ability.

### 1.2. Aim of the Review

To assess whether handwriting and typing share common brain circuits or whether brain activity differs while they are being performed, we reviewed studies on the neuroimaging of these two activities, alone or in combination. We chose to select only studies focusing on adult participants, since the brain is rapidly changing during childhood and adolescence and does not settle until age 24.

## 2. Materials and Methods

To identify possible neuroimaging signatures of handwriting and typing, we conducted the following search of the PubMed database, on December 5, 2024: (handwriting AND typewriting) OR (handwriting[ti] AND (neuroimaging[ti] OR fMRI[ti] OR (functional[ti] AND MRI[ti]) OR spectrosc*[ti] OR NIRS[ti] OR resonance[ti] OR electroencephalogr*[ti] OR EEG[ti] OR PET[ti] OR SPECT[ti] OR SPET[ti] OR photon[ti] OR proton[ti] OR positron[ti] OR magnetoenc*[ti] OR BOLD[ti] OR rCBF[ti])) OR ((keyboard[ti] OR typewrit*[ti]) AND (neuroimaging[ti] OR fMRI[ti] OR (functional[ti] AND MRI[ti]) OR spectrosc*[ti] OR NIRS[ti] OR resonance[ti] OR electroencephalogr*[ti] OR EEG[ti] OR PET[ti] OR SPECT[ti] OR SPET[ti] OR photon[ti] OR proton[ti] OR positron[ti] OR magnetoenc*[ti] OR BOLD[ti] OR rCBF[ti])). This search was intended to capture all aspects of brain activations or deactivations during hand- or typewriting in order to identify specific patterns of brain activation; so, it consisted of three different searches combined, one referring to both handwriting and typing together, and one for the neuroimaging of each writing subtype. Once gathered, all articles were downloaded and assessed for eligibility by consensus encounters in Delphi-style rounds in which all authors participated until full consensus was reached.

**Figure 1:**
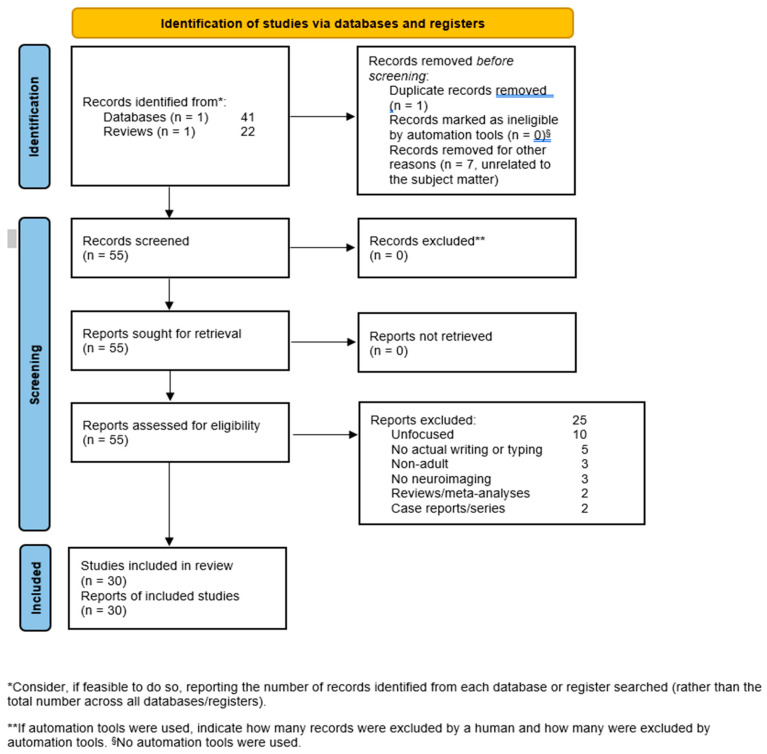
PRISMA flow diagram of our search with reasons for exclusion.

Studies were included that assessed human subjects while handwriting or while typing, using fMRI or EEG or other functional imaging techniques to allow the investigators to identify the loci of activation. Furthermore, given the evolution of writing modalities, future studies should explicitly indicate whether handwriting refers to traditional pen-and-paper writing or stylus-based writing on digital screens. As touchscreen and stylus input methods differ in haptic feedback and motor execution, this distinction would enhance the relevance and applicability of findings, particularly in the context of modern technology. All studies were excluded that did not provide data or adopted methods unfit for our purpose. We excluded opinion papers such as editorials, hypotheses, letters to the editor, and viewpoints, animal studies, reviews, meta-analyses, and guidelines collectively labelled as reviews, case reports, and case series, and all unfocused or unrelated articles emerging through our research strategy. Although reviews were not included among the eligible articles, we hand-searched their reference lists to identify studies that had possibly eluded our search. We did not include studies on children or adolescents, as their brains are still developing and have yet to reach their definitive state. Our results and decisions on eligibility are shown in Supplementary Table S1 and in Figure 1, where the PRISMA flow chart is displayed including reasons for exclusion (Figure 1). The locations of the included studies are presented in Supplementary Table S2.

## 3. Results

Our search of the PubMed database was conducted on 5 December 2024; it produced 41 records, to which further 22 were added that were obtained either serendipitously or through hand searching of the reference lists in reviews and meta-analyses, giving a total of 63 articles. Of these, 30 were eligible, while the rest were excluded for various reasons (Figure 1).

**Figure 2:**
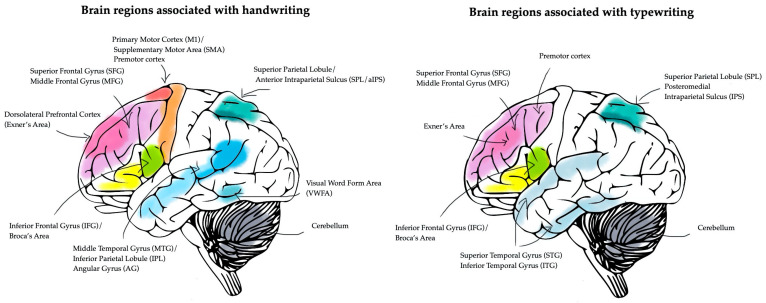
Neural correlates of handwriting and typewriting.

The results suggest that while handwriting primarily activates the motor cortex and visuospatial integration areas, typing predominantly engages linguistic processing and working memory circuits. Figure 2 compares the neural networks involved in handwriting and typing.

However, these differences are not always pronounced. A study by Planton et al. [5] on 16 right-handed individuals demonstrated that handwriting led to activations in the left intraparietal sulcus and premotor cortex, indicating involvement in motor control processes. Handwriting significantly activated the left fusiform area, which is crucial for visual word recognition. Methodological differences also influence the conclusions that have been drawn. Karimpour et al. [26] used a tablet with real-time visual feedback to monitor handwriting, revealing that cortical activation during handwriting was more widespread compared with studies using traditional pen-and-paper writing. Specifically, the use of a digital interface reduced involvement of the somatosensory cortex, suggesting that physical contact with paper might be a key factor in the cognitive processing of handwriting. These findings indicate that the differences between handwriting and typing are not solely dependent on motor execution but are also influenced by user experience, experimental methodology, and the type of writing device employed. The data reviewed suggest that handwriting and typing engage overlapping yet distinct neural networks (Table 1).

Studies by Longcamp et al. [22] and Palmis et al. [23] demonstrate that handwriting facilitates greater multisensory integration involving motor, parietal, and language-related cortical regions. Meanwhile, typing relies more heavily on executive and memory-related circuits, as evidenced by the findings of Purcell et al. [27] and Shah et al. (Table 2) [28]. However, the increasing adoption of digital writing technologies, such as stylus-based tablet input and touchscreen writing, may modify these patterns of brain activation.

Future studies should move beyond the traditional handwriting vs. typing dichotomy and consider the impact of emerging technologies, which may provide an experience more similar to traditional handwriting while retaining the advantages of digital text processing.

## 4. Discussion

This review synthesises existing evidence on the neural mechanisms of handwriting and typing, aiming to highlight both shared and distinct neural substrates. Handwriting and typing are distinct modes of written communication that engage different neural and cognitive processes, with significant implications for learning, memory, and brain development. A primary distinction between handwriting and typing lies in the differential activation of brain regions involved in motor control, sensory processing, and higher-order cognitive functions. These differences in neural engagement have profound implications for the cognitive processes associated with each mode of writing. Table 3 summarizes studies focusing on neural activation while handwriting.

Handwriting and typing are distinct activities that engage different motor and cognitive processes. Handwriting typically involves the use of one hand and focuses attention on a specific physical space, while typing requires the use of both hands and involves interaction with both the keyboard and the screen. These differences are reflected in the brain, as handwriting and typing activate distinct regions associated with motor control, sensory processing, and higher-order cognitive functions. Handwriting relies on sensorimotor coordination and tactile feedback, fostering a more intimate connection with the writing surface compared with the distanced interaction of typing. These unique characteristics lead to distinct learning processes during handwriting and typing, resulting in different learning outcomes.

Handwriting involves the complex coordination of fine motor skills, where each letter is individually formed through deliberate hand movements. This engages the sensorimotor cortex, which processes tactile feedback and motor control, as well as visual areas for letter recognition. The act of writing stimulates the brain to connect motor activities with cognitive processes, enhancing neural activity in areas associated with memory and language. Conversely, typing relies on repetitive finger movements over a keyboard. While it activates motor areas, it does not demand the intricate sensory–motor integration required by handwriting. The neural pathways activated during handwriting overlap significantly with those involved in reading and spelling, supporting literacy development. Studies using fMRI have shown that handwriting engages areas such as Broca’s area, which is critical for language production, as well as the parietal and temporal lobes, which support visual and auditory integration [17]. By comparison, typing activates fewer regions associated with language and relies more on procedural memory for key positioning (Table 4).

For young children, learning to write by hand has been linked to improved letter recognition and reading fluency, emphasizing its role in building foundational literacy skills [37]. The slower pace of handwriting allows more reflective and deliberate thinking, fostering creativity and critical analysis. Typing, while faster and more suited for brainstorming, often leads to a cognitive trade-off where the speed of transcription inhibits deeper thought. This difference may explain why handwriting is often preferred for tasks requiring problem-solving or synthesis of ideas.

Mangen and Velay [38] emphasized the concept of “haptic perception”, defined as a combination of tactile perception and voluntary movements. Haptic perception plays a crucial role in exploratory hand movements and object manipulation, making it highly relevant to the graphic gesture. The authors argue that writing transcends a purely mental process, necessitating the integration of visual, proprioceptive (haptic and kinesthetic), and tactile data, developing through two interconnected matrices: perceptual and graphomotor. When writing with a pen, the brain receives concurrent motor and sensory feedback, the latter arising from the fingers’ contact with the pen and paper. Conversely, novel technological tools for writing radically alter hand movements, consequently modifying haptic feedback and significantly impacting writing skills. Keyboard typing, while a motor activity, relies on the construction of a representational scheme mapping character shapes to key positions. However, since the same key can be activated by various finger movements, this mechanical process often disrupts the precise correspondence between a single gesture and the final product [26]. With the rapid advancement of technology, handwriting is being gradually replaced by typing. Children are increasingly learning to type on computers and smartphones before mastering handwriting. From an educational perspective, it is crucial to investigate when and to what extent the differences between these two writing modalities become significant, and what the short-term and long-term implications might be, both cognitively and socially.

These differences between handwriting and typing have profound implications for learning, creativity, and memory. Handwriting and typing share the same letter-based core processes: long-term phonological and orthographic memory, the semantic system, and the conversion of phonemes into graphemes and manual motor commands. Regarding the variances between these two writing modalities, several distinctions can be drawn. Firstly, from the perspective of the tool used, handwriting with pen and paper primarily involves one hand, engaging a predominantly left-lateralized neural network. Given the left hemisphere’s role in language processing, it can be inferred that handwriting induces greater neural activation related to letter production. However, typing requires the use of both hands and may thus rely on more interhemispheric neural activity.

Another key difference lies in the spatial and temporal demands of the two tasks. Handwriting requires focused attention on a limited space, encompassing a small portion of the paper and the pen tip, and it takes more time as each letter is formed through a completed movement. Typing involves attention to two distinct spaces: the keyboard for motor actions and the screen for visual feedback. It also requires less time, as typed letters appear almost instantaneously. However, the slower pace of handwriting may facilitate memorization of letters and words, leading to longer retention. The greater focus of attention involved in handwriting also contributes to a more intense level of cognitive processing [39].

In terms of sensorimotor experience, compared with typing, handwriting allows the writer to focus their attention on a single point in both space and time. This is because the graphic gesture unfolds in a concise manner within a very limited space, restricted to the pen tip and the trace of ink on the paper. Moreover, handwriting establishes a direct and exclusive relationship between the writer’s motor action and the resulting graphic output, involving a full-body and sensory experience.

Regarding motor programs, the learning process differs significantly between the two activities. As previously mentioned, handwriting requires the execution and control of a movement that fully defines the shape of a letter. Once learned, there is a unique correspondence between the letter and the movement that produces it, demanding a deeper level of attentional processing. Typing, on the other hand, also involves spatial learning, as individuals must construct a mental map of the keyboard to accurately locate and press each key. Unlike handwriting, the finger movement required to reach and press a key is simpler and more intuitive, lacking any graphomotor component. It has no direct relationship to the shape of the letter [16].

The absence of shape processing in typing hinders the development of the sensorimotor system. This is significant; excessive computer typing, as opposed to handwriting, results in reduced precision in hand-arm movements and a decline in other fine motor abilities over time [38].

Based on the sensory (visual and tactile) and motor experiences associated with handwriting, memory traces are formed and partially reactivated during retrieval. This perspective also applies to letter recognition; as discussed above, the coupling of motor action and sensory feedback during handwriting facilitates the development of this skill more than typing does. Therefore, from this standpoint, it is assumed that a single sensory modality is sufficient to activate the entire distributed network that was involved when the letter was initially memorized. Indeed, in four-year-old children who cannot name or write all the letters of the alphabet, handwriting training contributes to the specialization of the perceptual–motor neural network, enabling them to identify letters [40]. In this regard, it can be hypothesized that changing the motor conditions by using a typing-based method while children are learning to write would influence subsequent performance in letter memorization and processing.

### 4.1. Motor Control and Cognitive Engagement

Handwriting is a highly complex task that involves the integration of fine motor skills, visual processing, and cognitive functions related to memory and learning. It engages brain regions responsible for motor planning, such as the supplementary motor area (SMA) and posterior parietal cortex (PPC), as well as areas involved in visual and language processing, including the visual word form area (VWFA) and primary motor cortex (M1). The coordination of these diverse brain regions requires significant cognitive effort, which has been shown to facilitate deeper engagement with the material and enhance memory retention. For example, research by Longcamp et al. [22] and Siebner et al. [31] demonstrated that handwriting activates a broader network of motor-related and language-processing areas compared with typing, suggesting that the process of writing by hand is inherently more cognitively demanding. Through its requirements of fine motor execution and spatial awareness, handwriting appears to promote stronger connections between visual, motor, and cognitive functions, thereby reinforcing memory and learning. The work of Tremblay et al. [41] further supports this, indicating that handwriting enhances the ability to recognize and recall information due to its more demanding cognitive nature (Table 5).

In contrast, typing primarily engages the motor coordination of the fingers, a task that is less cognitively demanding compared with the fine motor control required for handwriting. Higashiyama et al. [35] utilized fMRI to compare the neural activation patterns of typing and handwriting and found that typing activated motor areas involved in automated, repetitive tasks but was associated with less engagement in areas responsible for complex motor planning. As a result, typing may foster more passive engagement with the material, with less integration between motor and cognitive functions.

Some authors have argued that the recognition of individual letters is enhanced when learning to write by hand rather than on a keyboard, as the fine motor activities involved in handwriting contribute to the recognition of the letters themselves. fMRI imaging studies have elucidated the mechanism underlying these differences. Longcamp and colleagues [50] demonstrated that the motor and premotor cortices are significantly more activated during the production of handwritten letters compared with printed ones. Similar results were obtained by Wamain et al. [51] through the study of event-related potentials (brain waves formed automatically in response to external stimuli) at the level of the occipital cortex (the seat of visual perception) in a task reflecting “motor familiarity” with the observed letters. These results also indicate that the motor information contained in handwritten letters is processed by the brain and that this processing depends on the activation state of the motor cortex corresponding to the limb used in writing. This suggests a more general principle whereby the observation and execution of an action facilitate its visual perception.

### 4.2. Cognitive Processing: Memory and Learning Outcomes

The cognitive implications of these differential activation patterns are particularly relevant to memory and learning. Several studies suggest that handwriting promotes superior memory retention compared with typing, a phenomenon that is probably associated with the more extensive neural engagement that handwriting demands. As Watanabe et al. [52] note, handwriting activates brain regions associated with episodic memory and deeper encoding processes. The effortful nature of handwriting, which requires the formation of each letter and the integration of visual, motor, and cognitive processes, enhances the brain’s ability to retain information over the long term.

Statistically, most studies on the relationship between handwriting and memory (including those conducted in Japan, Norway, and the United States) have shown that people remember information better when it is handwritten compared with when it is typed [50,53]. A pivotal study by Longcamp et al. [22] found that individuals who wrote characters by hand demonstrated better memory retention and faster recall than those who typed the same characters. This advantage was attributed to the greater cognitive load associated with handwriting, which promoted more effective encoding of information. Similarly, Barton et al. [34] observed that handwriting engaged the visual word form area (VWFA) to a greater extent than typing, further suggesting that handwriting leads to deeper processing of written information.

While typing is undeniably faster and more efficient, it appears to activate fewer neural circuits related to memory and learning. The automatic nature of typing, requiring less cognitive effort, may explain why typing is often less effective for tasks that require detailed processing and memory encoding. Barton et al. [32] and Tremblay et al. [41] suggest that the automaticity of typing reduces the level of neural engagement necessary for deeper cognitive processing, potentially leading to diminished memory retention compared with handwriting. Conflicting findings regarding handwriting and typing are likely to stem from differences in experimental design, participant populations, and imaging methodologies. For example, Nakamura et al. [20] used fMRI on Japanese participants and found that kanji handwriting activated the left posterior inferior temporal cortex, whereas kana typing activated frontal and parietal regions. This suggests that the complexity of writing and the language structure influence brain activation patterns. In contrast, Brownsett and Wise [48] studied English speakers via PET and found that handwriting activated bilateral inferior parietal lobules, irrespective of word complexity. Discrepancies may also arise from differences in task structure and control conditions. Askvik et al. [17] used high-density EEG and found that handwriting produced stronger event-related potentials (ERPs) than typing, but this effect was modulated by whether the task involved free writing or copying. Similarly, Bartoň et al. [34] demonstrated that letter writing produced higher functional connectivity between the visual word form area (VWFA) and motor cortices compared with simple dot writing, reinforcing the role of cognitive load in determining neural activation patterns. There may be a deeper motivation behind the avoidance of handwriting; young people may fear judgment from their peers, or they may be afraid of standing out. In fact, what is evident is the difficulty young people have in being themselves, a difficulty that leads to a desire for conformity at all costs, which then also becomes a mode of writing. Using only digital writing could therefore be a renunciation of authenticity, a limitation of one’s expressiveness, with consequences such as a thinning of personality and a loss of a personal and unique heritage [54]. While it is necessary for young people to learn to make their handwriting more legible, experts warn that it is essential to consider writing as the specific communication of the individual, a language whose alphabet is also constituted, especially in adolescence, by the groove left by the pen and by the arrangement of words in space, revealing how the person recognizes and represents themselves through their own use of signs. From this point of view, knowing how to write in cursive seems to be an achievement and not a starting point; it is an achievement of the “liberated” subject who expresses themselves in all their forms of intelligence, in their emotions, with their fears, because handwriting tells the story of the development of the individual subject in formation.

### 4.3. Functional Connectivity and Neural Integration

One of the more intriguing aspects of the comparison between handwriting and typing lies in the differences in functional connectivity between brain regions. Functional connectivity refers to the coordinated activation of different regions of the brain during a specific task. Studies suggest that handwriting promotes stronger and more widespread functional connectivity across a variety of brain regions, particularly those involved in motor control, sensory processing, and higher-order cognitive functions. This enhanced connectivity may explain why handwriting is associated with better learning and memory outcomes. Research by Van der Weel et al. [36] and Askvik et al. [17] demonstrates that handwriting tasks lead to increased theta band synchronization, which is linked to cognitive processing and memory consolidation. The broader neural engagement during handwriting tasks appears to foster more effective integration of sensory, motor, and cognitive information, enhancing memory encoding and retrieval. In contrast, typing tends to activate fewer regions and shows more limited connectivity between motor and cognitive areas, potentially leading to less robust consolidation of memory.

The manual tracing of cursive letters requires a complex motor pattern, as the form of each character must be continuously shaped to connect it to the following ones. This challenge does not arise when using electronic devices such as touchscreens, which rely on simpler and more repetitive gestures.

### 4.4. Educational Implications

The neural and cognitive distinctions between handwriting and typing have important implications for educational practices, particularly in terms of how students engage with written material. Given the enhanced cognitive engagement and memory retention associated with handwriting, it is vital to reconsider its role in the learning process, especially in an era where digital devices dominate educational environments. Research suggests that the act of handwriting may be particularly beneficial for tasks requiring critical thinking, analysis, and long-term retention of information, areas where typing may not offer the same advantages. As Askvik et al. [17] and Van der Weer and Van der Meer [36] suggest, the deeper neural engagement fostered by handwriting may contribute to improved learning outcomes, particularly for tasks that require complex cognitive processing. Van der Weer and Van der Meer [36] examined brain scans of 36 young adults enrolled in university who performed handwriting tasks. The authors observed that the entire brain was active during handwriting, while much smaller areas were active during typing. Additionally, the study reported that different parts of the brain activated during handwriting communicated through brain waves associated with learning. A substantial body of research has highlighted the role of alpha and theta oscillations in learning and memory, and Van der Weer and Van der Meer [36] found these oscillations to be active during handwriting but not during digital writing. In light of this evidence, educators and policymakers may consider prioritizing handwriting instruction in the curriculum, even as digital technologies continue to play an increasing role in education.

Mangen and Velay [38] highlight the importance of emphasizing the perceptual and sensorimotor components of reading and writing and their role in learning processes. Their findings suggest that the gestures involved in handwriting, unlike digital writing, contribute to the representation and memorization of characters, facilitating visual recognition. As grapheme recognition is a fundamental prerequisite for reading acquisition, a close relationship between handwriting and reading is implied. Writing and reading are inextricably linked and interdependent skills, as they result from the mental processes of encoding and decoding information. Children who have not learned to write well may experience difficulties in reading, comprehending the overall meaning of a text, understanding the context of words and phrases, and mastering spelling. Problems with handwriting, or a lack of automatic control, can hinder children’s progress, affect their notetaking abilities, and lead to concentration issues.

Graham et al. [55] demonstrated a strong correlation between handwriting proficiency and the quality of written texts. According to these researchers, teaching handwriting is associated with greater fluency of communication and higher-quality written output. Once the motor movements are automatized, students are better able to focus on the planning and organization of thought required for effective writing. Conversely, poor handwriting is often associated with spelling difficulties and lower performance in written tasks, in terms of both length and content [55,56]. Graham et al. [55] further observed that cursive writing promotes the development of self-control and is particularly effective in preventing letter reversals, a common problem in specific learning disorders. Indeed, when writing freely by hand, especially in cursive, not only must one plan and execute the action with greater precision, but the resulting output is also highly variable. This inherent variability in handwriting serves as a learning tool. The act of producing an imperfect letter can help children learn to decipher the variability of each letter, recognizing each symbol in its myriad forms. This is more effective for mental representation of the letter than repeatedly seeing the same identical symbol.

## 5. Conclusions

Writing is a complex phenomenon that requires diverse skills: perceiving the pen and paper, moving the writing instrument, and directing the movement through thought. Using a pen involves paying attention to motor aspects such as drawing letters legibly, controlling the pressure of the tip on the paper, following lines and spaces on the page, and coordinating thought, action, and vision. This multisensory integration underlies memory abilities. Moreover, handwriting involves a wide variety of supporting materials, including pens, pencils, or chalk on a blackboard, all of which offer different experiences and create new neural activations and skills.

Despite sharing similar central goals and processes, handwriting and typing differ significantly in terms of the tools used, spatiotemporal dimensions, motor programming, and fine motor development. Compared with handwriting, which requires more time and attention to learn, typing can be considered simpler and faster, as it enables the production of a more easily readable and homogeneous product in less time. However, focused attention and a longer processing time improve memory retention, and once automatic control of the graphic gesture is achieved, minimal cognitive effort is required. Moreover, the specific movements memorized when learning to write contribute to the visual recognition of graphic shapes and letters and secondarily also improve reading ability. Indeed, since the ability to recognize letters is widely recognized in the literature as the first phase of reading, improving it through writing may effectively influence how children read.

The comparison between handwriting and typing reveals important differences in their neural and cognitive impacts. Handwriting activates a broader network of brain regions involved in motor, sensory, and cognitive processing, contributing to deeper learning, enhanced memory retention, and more effective engagement with written material. Typing, while more efficient and automated, engages fewer neural circuits, resulting in more passive cognitive engagement. These findings suggest that despite the advantages of typing in terms of speed and convenience, handwriting remains an important tool for learning and memory retention, particularly in educational contexts.

We can consider handwriting and typing as distinct yet related modalities of written communication, each employing overlapping but specialized neural circuits. The unique demands of handwriting versus typing shape cognitive development and brain activity, rooted in our evolutionary history and present-day technological habits. The neuroscientific evidence highlights the cognitive advantages of handwriting in learning environments, particularly for younger learners. Handwriting and typewriting research opens new pathways for therapeutic interventions across various domains. Handwriting’s engagement with sensorimotor, cognitive, and memory-related networks makes it a valuable tool for cognitive rehabilitation in individuals with neurological conditions such as stroke, traumatic brain injury, or neurodegenerative diseases. Additionally, the activation of brain regions associated with emotional processing and self-awareness presents handwriting as a potential complementary strategy for addressing psychological conditions such as mood disorders and in psychotherapeutic interventions.

Digital technologies such as handwriting-simulating devices provide innovative solutions for patients with limited mobility, offering sensory feedback and promoting similar neural benefits to traditional handwriting. In aging populations, handwriting and typewriting can both stimulate neuroplasticity, potentially delaying cognitive decline. These tools also hold promise in educational and therapeutic contexts, enabling the design of hybrid approaches that combine the motor benefits of handwriting with the accessibility of typing, to create comprehensive rehabilitation strategies.

Future research should continue to explore the neural mechanisms underlying handwriting and typing, with a focus on how these differences may affect learning outcomes across different age groups, cultures, and contexts. Possible research questions include examining the cognitive load of handwriting versus typing in different age groups, the long-term effects of relying primarily on typing, and how individual differences such as handwriting style or typing proficiency influence cognitive outcomes. As digital technologies continue to evolve, it is crucial that we do not lose sight of the cognitive benefits associated with handwriting, particularly in the realms of education and memory consolidation.

It is important to deepen our understanding about how the advantages of handwriting might be integrated with digital technologies to create hybrid methods that combine the cognitive engagement of handwriting with the efficiency of typing. This approach could optimize learning outcomes and enhance writing practices in modern contexts.

## Figures and Tables

**Table 1:** Cerebral Areas Involved in Handwriting.

Cerebral Area	Function
Visual word form area (VWFA)	Stores letter shapes and orthographic knowledge.
Superior parietal lobule, anterior intraparietal sulcus (SPL/aIPS)	Visuospatial integration, motor planning, and letter-specific motor programs.
Dorsolateral prefrontal cortex (Exner’s area)	Translates letter representations into motor commands.
Primary motor cortex (M1) and supplementary motor area (SMA)	Executes and coordinates motor commands.
Inferior frontal gyrus (IFG), Broca’s area	Phoneme-to-grapheme conversion, crucial in phonological agraphia.
Middle frontal gyrus (MFG) and superior frontal gyrus (SFG)	Higher-level motor control and selection of well-practiced graphic patterns.
Cuneus/precuneus	Visual processing and attention.
Middle temporal gyrus (MTG) and angular gyrus (AG)	Language processing and integration of phonological and orthographic representations.
Ventral precentral gyrus and inferior parietal lobule (IPL)	Specialized for writing movements.
Sensorimotor striatum (smStr)	Integrates visual letter forms with motor execution.
Basal ganglia (striatum, putamen, pallidum, caudate nucleus)	Fine-tunes writing kinematics, motor sequence learning, and inhibition of competing actions.
Thalamus	Relays motor and sensory information.
Cerebellum	Adjusts movement precision, coordination, and timing.

**Table 2:** Cerebral Areas Involved in Typing.

Cerebral Area	Function
Inferior frontal gyrus (IFG), Broca’s area	Supports phoneme-to-grapheme conversion, spelling, and lexical retrieval.
Middle frontal gyrus (MFG), superior frontal gyrus (SFG)	Involved in higher-level motor planning and executive functions related to typing.
Intraparietal sulcus (IPS), superior parietal lobule (SPL)	Engages spatial processing and attention control, supporting key-to-finger mappings in typewriting.
Inferior temporal gyrus (ITG), fusiform gyrus (FuG)	Plays a role in orthographic processing and letter recognition.
Superior temporal gyrus (STG)	Involved in auditory and phonological processing during spelling tasks.
Right supramarginal gyrus (BA 40)	Engaged in spatial learning and attentional shifts when typing characters.
Posteromedial intraparietal sulcus (IPS)	Specific to typing, linked to fine motor control.
Basal ganglia (striatum)	Plays a role in procedural learning and automation of keypress sequences.
Thalamus	Relays motor and sensory information, but with reduced engagement compared with handwriting.
Cerebellum	Contributes to motor learning and coordination of typing movements.

**Table 3:** Summary of included studies on the neuroimaging of handwriting.

Study	Population	Technique	Conclusion/Observations
Petrides et al., 1995 [29]	11 ♂ r-h prtcs, $\bar{x}$ = 24, age range 18–32 yrs.	PET, H215° rCBF, 1.5T MRI co-registration on T&TSA.	VLPFC is involved in the strategic retrieval of verbal episodic information, while the DLPFC is activated during tasks requiring mnemonic output monitoring, and the SMCx and posterior PCx are activated during writing, suggesting distinct functional specializations within the frontal Cx.
Katanoda et al., 2001 [30]	17 r-h ♂ healthy prtcs, age range 19–31 yrs, with normal or corrected-to-normal vision.	fMRI, 1.5T, T2*-weighted BOLD aligned with T1-weighted scans for anatomic localization.	The results differentiate writing from other linguistic and motor activities.
Siebner et al., 2001 [31]	11 healthy r-h prtcs (2 ♀, 9 ♂; $\bar{x}$ age 42 yrs, range 26–58 yrs) vs. 11 healthy l-h converted to right handwriting (4 ♀, 7 ♂; $\bar{x}$ age 47 yrs, range 34–64 yrs) vs. controls, 6 consistent l-h, not converted to right hand writing (1 ♀, 5 ♂; $\bar{x}$ age 32 yrs, range 25–55 yrs).	PET, H215°-rCBF; analysis through SPM implemented in PRO-MATLAB environment normalized on MNI template; location of peak activation with T&TSA.	Writing elicited activation patterns in consistent r-h prtcs in the dominant (contralateral) hemisphere. Stronger activation of the right lateral PFC in converted l-h compared with more prominent activation in the left dorsal and ventral PFC during right-hand writing in r-h might reflect adaptation, i.e., more activation is needed to support a “corrected” behavior.
Beeson et al., 2003 [18]	12 healthy r-h adults (6 ♀; 6 ♂) age range 20- 53 yrs, $\bar{x}$ age = 38.4 yrs, English speakers.	fMRI, 1.5T, T2*-weighted BOLD aligned with T1-weighted scans to localize areas according to MNI-SPM99.	This study contradicts the long-proposed notion that orthographic representations for familiar words are stored in the dominant (left) AG (BA39) in the form of visual images.
Roux et al., 2009 [19]	12 > 63 ± 19.18% r-h ($\bar{x}$ age 22 ± 3.88 yrs and 12 > 69 ± 18.91% l-h healthy volunteers ($\bar{x}$ age 25 ± 5.05). Sex of volunteers not specified.	fMRI 1.5T; EPI sequenced images normalized on T&TSA template and analyzed with MATLAB 7 using SPM5.	An area located close to the superior frontal sulcus in BA6, anterior to the hand primary motor area, is selectively involved in handwritten word production. Handwriting-related activation in BA6 was bilateral in r-h prtcs but slightly ↑ in the dominant hemisphere.
Barton et al., 2010 [32]	13 healthy r-h English-literate individuals (5 ♀, 8 ♂; $\bar{x}$ age 28.1 ± 4.0 yrs, range 22–35 yrs) with normal or corrected-to-normal visual acuity.	fMRI 3T, T2-weighted functional images with EPI sequences; ROI approach combined with whole-brain analysis; images plotted on T&TSA.	Abstract word representations might emerge at higher processing levels. The VWFA is primarily involved in perceptual text analysis, particularly handwriting, rather than abstract linguistic processing and is sensitive to handwriting, supporting a primary role in perceptual rather than linguistic processing. Findings challenge the notion of the VWFA as exclusively dedicated to linguistic functions, emphasizing its contribution to visual and stylistic text analysis.
Segal & Petrides, 2012 [21]	Nine healthy r-h bilingual speakers (English–French or German or Chinese, six ♂, three ♀; $\bar{x}$ age 26 ±3.72 yrs).	fMRI, 1.5T, T2*-weighted BOLD aligned with T1-weighted scans to map according to the MNI co-ordinates.	PE area in the SPL of the language-dominant hemisphere is a critical high-level motor control region for writing. This area acts as an interface between cortical motor and language regions, coordinating hand actions required for producing written language.
Longcamp et al., 2014 [33]	18 native French speakers (11 ♀, 7 ♂; $\bar{x}$ age 24 yrs, range 18–35 yrs), with normal audition and normal or corrected-to-normal vision.	fMRI 3T, T1-weighted MP-RAGE sequence superimposed on T2*-weighted EPI, analyzed with SPM8 on MNI template.	Observed differences between letters and digits were not related to low-level kinematic features. Writing engages an extended motor-perceptual network. Only two very restricted areas discriminate between letters and numbers. The dorsal preMCx is a fundamental brain network node for handwriting.
Planton et al., 2017 [5]	16 healthy r-h native French (8 ♂, 8 ♀; $\bar{x}$ age 25.3 ± 6.0 yrs).	fMRI 3T EPI sequences normalized and realigned on MNI template and analyzed through SPM12b; VOI approach	The ventral part of the precentral gyrus and the left IPL show a preference for writing, suggesting specialization. Writing and oral spelling overlap with traditional language areas like left IFG (Broca’s area) and STG. Writing activates additional motor and visuospatial regions not involved in oral spelling. Results support a hierarchical model in which regions specific to writing overlap with more general circuits used for motor control and language. Findings are relevant to understanding writing disorders (e.g., dysgraphia) and designing therapeutic interventions.
Karimpour et al., 2018 [26]	12 r-h healthy Canadian adult graduates (5 ♂, 7 ♀; $\bar{x}$ age 25).	fMRI 3T T2*-weighted BOLD analyzed with SPM normalized on MNI template.	Behavioral results confirm that IAT metrics successfully discriminate performance differences across tasks. VPMc regions have a role in guiding hand movements. Variations in brain activity across the three tasks reflected differing cognitive processing demands.
Bartoň et al., 2020[34]	20 r-h healthy volunteers (9 ♀, 11 ♂; $\bar{x}$ age 23 ± 2.4 yrs)	fMRI 1.5T BOLD, EPI sequencing T1-weighted MRI analysed through SPM12 with normalization on the MNI template. ROI approach	The striatum plays a crucial role in integrating stored letter-shape representations with motor planning and execution processes during handwriting. A writing-specific cortico-striatal network comprises the VWFA, SPL/anterior IPS, striatum, preMCx/Exner’s area, primary motor cortex, and SMA. The striatum is important for integrating information about letter shapes, selecting/retrieving abstract motor programs, and generating concrete motor commands

Abbreviations: AG, angular gyrus; BA, Brodmann area (s); Cx, cortex; DLPFC, dorsolateral prefrontal cortex; EPI, echo-planar imaging; fMRI, functional magnetic resonance imaging; hd, high-density; IAT, in-air time; IFG, inferior frontal gyrus; IPL, inferior parietal lobule; IPS, intraparietal sulcus; MATLAB, a proprietary multi-paradigm programming language and numeric computing environment developed by MathWorks (MathWorks Laboratory); MNI, Montréal Neurological Institute; MP-RAGE, magnetization-prepared rapid gradient echo; PCx, parietal cortex; PET, positron emission tomography; PFC, prefrontal cortex; PreMCx, premotor cortex; prtc(s), participant(s); rCBF, regional cerebral blood flow; r-h, right-handed; ROI, region of interest; SMA, supplementary motor area; SMCx, sensorimotor cortex; SPL, superior parietal lobule; SPM, statistical parametric mapping; STG, superior temporal gyrus; T, Tesla; T&TSA, Talairach–Tournoux stereotaxic atlas; VLPFC, ventrolateral prefrontal cortex; VOI, volume of interest; VPMc, ventral premotor cortex; VWFA, visual word form area; wk (s), week (s); WT, writing task; $\bar{x}$, mean; yr (s), year (s); ×, for, per; ±, SD, standard deviation; ↓, decrease, decreased, lower, diminution, worsening; ↑, increase, augmentation, elevation, improvement, greater; →, followed by, subsequently; ♀, female; ♂, male.

**Table 4:** Summary of studies on the neuroimaging of typewriting and differences between handwriting and typewriting.

Study	Population	Technique	Conclusion/Observations
Purcell et al., 2011 [27]	17 healthy r-h English speakers (7 ♂, 10 ♀; $\bar{x}$ age 23.2 yrs, range 18–27 yrs).	fMRI, 3T; EPI sequences plus T1-weighted scans; images analyzed with SPM5 and normalized according to MNI.	Typewriting activates a spelling network that is active conjointly with reading. The typewriting spelling includes a region near Exner’s area, which is shared by handwriting. Lateral IFG activation associated with spelling was ˃ reading, while in the medial portion there was no significant difference spelling vs. reading.
Higashiyama et al., 2015 [35]	16 healthy r-h native Japanese speakers, skillful typists (9 ♂, 7 ♀; $\bar{x}$ age 27.8 ± 3.1 yrs, range 23–34 yrs).	fMRI 3T, T1-weighted images MP-RAGE sequences analyzed voxel-wise with FWE in MNI space using SPM8 implemented in MATLAB 7.7.0.	The brain regions known as the “writing centre” are the same for typing. Scan comparisons of typing and writing showed the left posteromedial IPS to be typing-specific. Activity in the left MFG/SFG was more rostral in the typing than in the writing task, pointing to differences in motor planning.
Askvik et al., 2020 [17]	12 r-h healthy school-aged children (four ♂, eight ♀; $\bar{x}$ age 11.83 ± 0.39 yrs); 12 r-h healthy young adults (six ♂, six ♀; $\bar{x}$ age 23.58 ± 2.02 yrs).	hd EEG, 256 channel with electrodes distributed over scalp.	Handwriting and drawing are associated with memory and learning processes; there is different cognitive engagement between handwriting and drawing vs. typewriting. Findings underscore the importance of handwriting and drawing activities in early childhood education. Sensorimotor integration and fine motor skills involved in these activities contribute to the development of neural networks essential for learning and cognitive development.
Van der Weel and Van der Meer, 2024 [36]	36 r-h prtcs in their early twenties; sex not specified.	hd EEG, 256 channel with electrodes distributed over scalp.	↑ FC between the various brain regions seems to be linked to the specific sensorimotor processes that are typical in handwriting. Spatial temporal patterns from visual and proprioceptive information during handwriting movements have a beneficial impact on the brain’s FC patterns related to learning and remembering.

Abbreviations: EEG, electroencephalogram, electroencephalography; EPI, echo-planar imaging; FC, functional connectivity; fMRI, functional magnetic resonance imaging; FEW, family-wise error; hd, high-density; IFG, inferior frontal gyrus; IPS, intraparietal sulcus; MATLAB, a proprietary multi-paradigm programming language and nueric computing environment developed by MathWorks (MathWorks Laboratory); MFG, middle frontal gyrus; MNI, Montréal Neurological Institute; MP-RAGE, magnetization-prepared rapid gradient echo; r-h, right-handed; SFG, superior frontal gyrus; T, Tesla; wk (s), week (s); mean; yr (s), year (s); ×, for, per; ±, SD, standard deviation; ↓, decrease, decreased, lower, diminution, worsening; ↑, increase, augmentation, elevation, improvement, greater; →, followed by, subsequently; ♀, female; ♂, male.

**Table 5:** Summary of studies on the neuroimaging of handwriting and typewriting, focusing on sensorimotor control, cognitive engagement, and practice implications.

Study	Population	Technique	Conclusion/Observations
Seitz et al., 1997 [42]	Eight healthy r-h Caucasian medical students (four ♂, four ♀, $\bar{x}$ age 28 ± 3 yrs) with normal or corrected visual acuity.	PET, [^15^O]butanol rCBF, 1.5T MRI co-registration on T&TSA.	The PCx engages in motor coordination and learning of new movement patterns associated with the automatic processing of previously consolidated movements. There are two subsystems, one engaged in controlled processing around the anterior IPS and another in posterior PCx during automatic execution. During mental imagery, both subsystems are simultaneously active.
Nakamura et al., 2000 [20]	10 healthy r-h students at Kyoto University, native Japanese speakers (6 ♂, 4 ♀, age range 20–25 yrs).	fMRI, 1.5T images analyzed through SPM96.	Evidence suggests that the left PITCx plays a central role in the retrieval of visual engrams of letters. During the act of writing, there is no need for conscious effort to recall the visual images of letters or words to be written, as the sequential visuomotor skill proceeds automatically due to the presence of a specialized neural sub-system for retrieving visual graphic forms.
Matsuo et al., 2001 [43]	12 r-h (1 ambidextrous) healthy Japanese (8 ♂, 4 ♀, age range 22–29 yrs).	fMRI 3T, T2-weighted images overlapping with fMRI; ROI analysis with SPM96 using T&TSA MR normalization.	Activation in higher motor areas may be induced by cognitive components related to motor function when performing visuospatial language tasks.
Siebner et al., 2001 [31]	10 healthy r-h prtcs (8 ♂, 2 ♀; $\bar{x}$ age 41.3 ± 10.9 yrs).	PET, H_2_^15^O-rCBF normalized on T&TSA MRs	A set of cortical and subcortical brain regions is involved in the processing of movements associated with SC-L writing. FO-L handwriting is achieved by optimized cooperation of the manual sensorimotor network rather than by selective activation of a distinct network component. The functional activation pattern during handwriting is not influenced by different levels of motor learning.
Nakamura et al., 2002 [44]	Nine healthy r-h volunteers (age range 21–30 yrs), native Japanese speakers.	fMRI, 1.5T images analyzed through SPM96.	Contrary to the traditional view, the obtained functional imaging evidence indicated that in normal people, the neural systems for processing the two different scripts are separable only functionally, rather than anatomically.
Omura et al., 2004 [45]	15 healthy r-h adults (9 ♂, 6 ♀), age range 18–39 yrs ($\bar{x}$ age 25.7 ± 6.4), Japanese speakers.	fMRI, 1.5T, T2*-weighted BOLD aligned with T1-weighted; areas defined with MNI-SPM99.	Findings suggest that the left premotor area transfers phonemic representations to graphemic motor output to create letters in writing to dictation. The left STG is thought to transform auditory information into phonetic (auditory) and/or graphic (visual) representations of letters.
Rektor et al., 2006 [46]	10 healthy r-h adult Czech speakers (2 ♀, 8 ♂) age range 20–25 yrs, $\bar{x}$ age 23.5 ± 1.28.	fMRI, 1.5T, T1-weighted scans fitted on fMRI images to localize areas using SPM99.	The right hemisphere is dominant for tasks requiring manipulation in space. It is possible that the activation of this region is linked with the spatial dimension of the writing. Right-sided PCx may play an important role in the elemental mechanism of writing.
Harrington et al., 2007 [47]	11 healthy r-h adults (5 ♂, 6 ♀); $\bar{x}$ age 37.1 yrs, range 22–61 yrs, of whom 6 (2 ♂, 4 ♀; $\bar{x}$ age 39.0 yrs, range 26–54 yrs) participated in Experiment 2.	fMRI 1.5T, T1-weighted images EPI sequences analyzed through AFNI AlphaSim normalized on T&TSA; ROI approach.	Mental imagery of motion recruits the same Cx areas used in motor performance. The differences in activation reflect the use of distinct brain pathways for symbolic (writing) vs. visuo-spatial (drawing) processes.
Longcamp et al., 2008 [22]	12 r-h prtcs (6 ♂, 6 ♀; $\bar{x}$ age 26 ± 3 yrs).	fMRI 3T, T2*-weighted EPI sequence superimposed on T1-weighted images; analysis with SPM2 normalized on MNI template; significant clusters identified with T&TSA.	Better recognition of new characters when written compared with typed. Handwriting memory facilitated discrimination between characters and their mirror images for longer periods than typewriting memory. Via fMRI, different neural circuits were found to underlie differences in recognition performance between hand- and typewritten characters, supported by left-sided lateralization of activations.
Tremblay et al., 2008 [41]	ST: Six healthy r-h English speakers (four ♀, two ♂; $\bar{x}$ age 26 ± 4.91 yrs). NST: Six healthy r-h English speakers (three ♀, three ♂; $\bar{x}$ age 22.7 ± 2.27 yrs).	EEG 64-pin active Ag-AgCl electrodes, EMG of the orbicularis oris muscle with two flat electrodes placed above upper and lower lip.	β ERD patterns showed a closer relation to response selection. In contrast, α ERD patterns were more closely related to response onset, perhaps reflecting attentional demands overseeing execuction of motor response. ERD patterns for the speech and keyboard-pressing tasks were very similar across the frequency bands, suggesting that the orofacial and finger motor systems used similar underlying neural mechanisms.
Brownsett & Wise, 2010 [48]	Three healthy r-h (seven ♂, six ♀; $\bar{x}$ age 51.7 yrs, range 40–70 yrs).	PET, H_2_^15^O rCBF; images analyzed with SPM2.	Extensive parietal activity is associated with the planning, execution, and monitoring of writing, even for only a single letter. The PL contributes little to the planning, execution, and monitoring of articulation during normal spoken language production. Parietal activity for amodal linguistic or mnemonic processing is confined to the left AG.
Shah et al., 2013 [28]	28 r-h prtcs (14 ♀, 14 ♂; $\bar{x}$ age 24.0 ± 1.9 yrs) in-experienced in creative writing, native German speakers.	fMRI 3T, BOLD, T1-weighted, EPI, images analysed using SPM5 run on MATLAB 7.4 normalized to MNI.	Creative writing and copying shared many brain activations and correlated with creativity indexes; hence, copying is not devoid of creative processes and their related brain activations, mostly the SFG–temporopolar focus. Brainstorming involved novel and original idea generation and composition of the story concept through recruiting fronto-parieto-temporal circuits. Some PreMCx activity may have been involved in preparatory processes for writing activity. Creative writing combines handwriting with cognitive processes associated with writing, such as episodic memory, semantic integration, and text production, linked to activation of a left fronto-temporal network including left IFG (BA45) and left temporopolar Cx (BA38).
Bisio et al., 2016 [49]	Enrolled 44 r-h prtcs, 22 (12 ♀, 10 ♂; $\bar{x}$ age 25 ± 5.6 years) in Ex-periment 1, 22 (14 ♀, 8 ♂; $\bar{x}$ age 24.2 ± 6.1 years) in Experiment 2, of whom 7 underwent fMRI scanning (sex and $\bar{x}$ age not specified).	fMRI 1.5T, T2*-weighted sin-gle-shot EPI sequences normal-ised on MNI template analysed with SPM12.	The tablet reliably captured kinematic data across time, including fairly predictably under constrained MR conditions. fMRI identified activations in brain regions purportedly associated with handwriting in healthy people.
Palmis et al., 2019 [23]	25 r-h native French speakers ($\bar{x}$ age 24, range 19–37); sex not specified.	fMRI 3T, T2* BOLD and T1 MR superimposition analyzed through SPM12. ROI.	Core regions of the written language network, like left IFG and FuG, are sensitive to orthographic irregularities during writing. Motor-related regions, like SFG and SPL, exhibited ↑ activation for irregular words. Findings support dynamic interactive and parallel orthographic and motor processes during handwriting.
Boyraz et al., 2021 [37]	26 r-h ♀ with MDD, $\bar{x}$ age range 18–50 yrs.	rs-fMRI 1.5T, T1-weighted MP-RAGE sequence for ROI analysis with CONN software using MATLAB-based SPM.	Adding NHE to standard treatment may be associated with modest improvements in symptoms of depression, despite significant differences between NHE and non-NHE in their FC changes.

Abbreviations: AFNI, analysis of functional neuroimages; AG, angular gyrus; Cx, cortex; EPI, echo-planar imaging; ERD, event-related desynchronization; FC, functional connectivity; fMRI, functional magnetic resonance imaging; FO-L, fast open loop; FuG, fusiform gyrus; IFG, inferior frontal gyrus; MATLAB, a proprietary multi-paradigm programming language and numeric computing environment developed by MathWorks (MathWorks Laboratory); MNI, Montréal Neurological Institute; MP-RAGE, magnetization-prepared rapid gradient echo; MR, magnetic resonance; NHE, non-dominant hand exercise; PCx, parietal cortex; PET, positron emission tomography; PITCx, posteroinferior temporal cortex; PreMCx, premotor cortex; prtc (s), participant (s); rCBF, regional cerebral blood flow; r-h, right-handed; rs, resting state; ROI, regions of interest; SC-L, slow close loop; STG, superior temporal gyrus; T&TSA, Talair-ach-Tournoux stereotaxic atlas; wk (s), week (s); mean; yr (s), year (s); ×, for, per; ±, SD, standard deviation; ↓, decrease, decreased, lower, diminution, worsening; ↑, increase, augmentation, elevation, improvement, greater; →, followed by, subsequently; ♀, female; ♂, male.

## Data Availability

Data are contained in the article and cited articles.

## Supplementary Materials

The following supporting information can be downloaded at https://www.mdpi.com/article/10.3390/life15030345/s1: Table S1: Search strategy on PubMed and other sources (serendipity or reference lists of reviews) and study labelling. Table S2: Study locations.
