# Supplementary material for: The Neuroscience Behind Writing: Handwriting vs. Typing—Who Wins the Battle?
Source: Life (Basel). 2025 Feb 22;15(3):345. doi: 10.3390/life15030345 (PMC11943480; doi:10.3390/life15030345)
Supplement: Supplementary file 1 [file life-15-00345-s001.zip › life-3428671-supplementary.pdf]

Supplementary Table S1. Search strategy on PubMed and other sources (serendipity or reference lists of reviews) and study labelling.

(handwriting AND typewriting) OR (handwriting[ti] AND (neuroimaging[ti] OR fMRI[ti] OR (functional[ti] AND MRI[ti]) OR spectrosc*[ti] OR NIRS[ti] OR resonance[ti] OR electroencephalogr*[ti] OR EEG[ti] OR PET[ti] OR SPECT[ti] OR SPET[ti] OR photon[ti] OR proton[ti] OR positron[ti] OR magnetoenc*[ti] OR BOLD[ti] OR rCBF[ti])) OR ((keyboard[ti] OR typewrit*[ti]) AND (neuroimaging[ti] OR fMRI[ti] OR (functional[ti] AND MRI[ti]) OR spectrosc*[ti] OR NIRS[ti] OR resonance[ti] OR electroencephalogr*[ti] OR EEG[ti] OR PET[ti] OR SPECT[ti] OR SPET[ti] OR photon[ti] OR proton[ti] OR positron[ti] OR magnetoenc*[ti] OR BOLD[ti] OR rCBF[ti])) PubMed 6.12.2024 → 41 results

1	Harrison WR. Some forensic aspects of handwriting and typewriting. Med Leg J. 1953;21(1):2-9. doi: 10.1177/002581725302100102.	Unrelated
2	Provins KA, Glencross DJ. Handwriting, typewriting and handedness. Q J Exp Psychol. 1968;20(3):282-9. doi: 10.1080/14640746808400162.	Unfocused
3	Seron X, Deloche G, Moulard G, Rousselle M. A computer-based therapy for the treatment of aphasic subjects with writing disorders. J Speech Hear Disord. 1980;45(1):45-58. doi: 10.1044/jshd.4501.45.	Unfocused
4	Siegel LS, Feldman W. Nondyslexic children with combined writing and arithmetic learning disabilities. Clin Pediatr (Phila). 1983;22(4):241-4. doi: 10.1177/000992288302200401.	Unrelated
5	Boyle M, Canter GJ. Neuropsychological analysis of a typewriting disturbance following cerebral damage. Brain Lang. 1987;30(1):147-64. doi: 10.1016/0093-934x(87)90034-4.	No NI
6	Oehmichen M, von Kortzfleisch D, Hegner B. Rasterelektronenmikroskopische Untersuchungen zur Analyse der Strichkreuzung [Scanning electron microscopy studies for the analysis of mixed script]. Beitr Gerichtl Med. 1989;47:203-6. German.	Review
7	Oehmichen M, von Kortzfleisch D, Hegner B. Die Schriftkreuzung im Rasterelektronenmikroskop (REM) [Script crossing in scanning electron microscopy]. Z Rechtsmed. 1989;102(4):219-30. German. doi: 10.1007/BF00201316.	Unrelated
8	Tei H, Soma Y, Maruyama S. Right unilateral agraphia following callosal infarction in a left-hander. Eur Neurol. 1994;34(3):168-72. doi: 10.1159/000117032.	Case
9	Umeda T, Inamura K, Inamoto K, Ikezoe J, Kozuka T, Kawase I, Fujii Y, Karasawa H. Development and evaluation of oral reporting system for PACS. Comput Methods Programs Biomed. 1994;43(1-2):115-23. doi: 10.1016/0169-2607(94)90196-1.	Unrelated
10	Matsuo K, Kato C, Tanaka S, Sugio T, Matsuzawa M, Inui T, Moriya T, Glover GH, Nakai T. Visual language and handwriting movement: functional magnetic resonance imaging at 3 tesla during generation of ideographic characters. Brain Res Bull. 2001;55(4):549-54. doi: 10.1016/s0361-9230(01)00564-0.	**Included**
11	Siebner HR, Limmer C, Peinemann A, Bartenstein P, Drzezga A, Conrad B. Brain correlates of fast and slow handwriting in humans: a PET-performance correlation analysis. Eur J Neurosci. 2001;14(4):726-36. doi: 10.1046/j.0953-816x.2001.01694.x.	**Included**
12	Siebner HR, Limmer C, Peinemann A, Drzezga A, Bloem BR, Schwaiger M, Conrad B. Long-term consequences of switching handedness: a positron emission tomography study on handwriting in "converted" left-handers. J Neurosci. 2002;22(7):2816-25. doi: 10.1523/JNEUROSCI.22-07-02816.2002.	**Included**
13	Chaikovsky A, Brown S, David LS, Balman A, Barzovski A. Color separation of signature and stamp inks to facilitate handwriting examination. J Forensic Sci. 2003;48(6):1396-405. doi: 10.1520/JFS2002425.	Unrelated
14	Obermaier B, Müller GR, Pfurtscheller G. "Virtual keyboard" controlled by spontaneous EEG activity. IEEE Trans Neural Syst Rehabil Eng. 2003;11(4):422-6. doi: 10.1109/TNSRE.2003.816866.	No NI
15	Scherer R, Müller GR, Neuper C, Graimann B, Pfurtscheller G. An asynchronously controlled EEG-based virtual keyboard: improvement of the spelling rate. IEEE Trans Biomed Eng. 2004;51(6):979-84. doi: 10.1109/TBME.2004.827062.	No NI
16	Shafer-Crane GA, Meyer RA, Schlinger MC, Bennett DL, Robinson KK, Rechtien JJ. Effect of occupational keyboard typing on magnetic resonance imaging of the median nerve in subjects with and without symptoms of carpal tunnel syndrome. Am J Phys Med Rehabil. 2005;84(4):258-66. doi: 10.1097/01.phm.0000156897.44954.e2.	Unfocused
17	Kačar A, Svetel M, Jović J, Pekmezović T, Kostić VS. Графоспазам—клиничка презентација, етиологија и ток болести: анализа 30 случајева Grafospazam—klinicka prezentacija, etiologija i tok bolesti: analiza 30 slucajeva [Graphospasm—clinical presentation, etiology and the course of disease: analysis of 30 cases]. Srp Arh Celok Lek. 2004;132(11-12):385-9. Serbian. doi: 10.2298/sarh0412385k.	Unfocused
18	Van Gemmert AW, Teulings HL. Advances in graphonomics: studies on fine motor control, its development and disorders. Hum Mov Sci. 2006;25(4-5):447-53. doi: 10.1016/j.humov.2006.07.002. Epub 2006 Oct 5.	Unfocused
19	Longcamp M, Boucard C, Gilhodes JC, Anton JL, Roth M, Nazarian B, Velay JL. Learning through hand- or typewriting influences visual recognition of new graphic shapes: behavioral and functional imaging evidence. J Cogn Neurosci. 2008;20(5):802-15. doi: 10.1162/jocn.2008.20504.	**Included**
20	Tremblay P, Shiller DM, Gracco VL. On the time-course and frequency selectivity of the EEG for different modes of response selection: evidence from speech production and keyboard pressing. Clin Neurophysiol. 2008;119(1):88-99. doi: 10.1016/j.clinph.2007.09.063.	**Included**
21	Barton JJ, Fox CJ, Sekunova A, Iaria G. Encoding in the visual word form area: an fMRI adaptation study of words versus handwriting. J Cogn Neurosci. 2010;22(8):1649-61. doi: 10.1162/jocn.2009.21286.	**Included**
22	Magrassi L, Bongetta D, Bianchini S, Berardesca M, Arienta C. Central and peripheral components of writing critically depend on a defined area of the dominant superior parietal gyrus. Brain Res. 2010;1346:145-54. doi: 10.1016/j.brainres.2010.05.046. Epub 2010 May 24.	Case
23	Orhan U, Hild KE 2nd, Erdogmus D, Roark B, Oken B, Fried-Oken M. RSVP Keyboard: An EEG based typing interface. Proc IEEE Int Conf Acoust Speech Signal Process. 2012:10.1109/ICASSP.2012.6287966. doi: 10.1109/ICASSP.2012.6287966.	Unfocused
24	Planton S, Jucla M, Roux FE, Démonet JF. The "handwriting brain": a meta- analysis of neuroimaging studies of motor versus orthographic processes. Cortex. 2013;49(10):2772-87. doi: 10.1016/j.cortex.2013.05.011. Epub 2013 Jun 12.	Review
25	Pfordresher PQ, Mantell JT, Brown S, Zivadinov R, Cox JL. Brain responses to altered auditory feedback during musical keyboard production: an fMRI study. Brain Res. 2014;1556:28-37. doi: 10.1016/j.brainres.2014.02.004. Epub 2014 Feb 7.	Unfocused
26	Gimenez P, Bugescu N, Black JM, Hancock R, Pugh K, Nagamine M, Kutner E, Mazaika P, Hendren R, McCandliss BD, Hoeft F. Neuroimaging correlates of handwriting quality as children learn to read and write. Front Hum Neurosci. 2014;8:155. doi: 10.3389/fnhum.2014.00155.	Non-adult
27	Higashiyama Y, Takeda K, Someya Y, Kuroiwa Y, Tanaka F. The neural basis of typewriting: A functional MRI study. PLoS One. 2015;10(7):e0134131. doi: 10.1371/journal.pone.0134131. Erratum in: PLoS One. 2015;10(8):e0137265. doi: 10.1371/journal.pone.0137265.	**Included**
28	Higashiyama Y, Takeda K, Someya Y, Kuroiwa Y, Tanaka F. Correction: The neural basis of typewriting: A functional MRI study. PLoS One. 2015;10(8):e0137265. doi: 10.1371/journal.pone.0137265. Erratum for: PLoS One. 2015;10(7):e0134131. doi: 10.1371/journal.pone.0134131.	Duplicate
29	Kiefer M, Schuler S, Mayer C, Trumpp NM, Hille K, Sachse S. Handwriting or Typewriting? The influence of pen- or keyboard-based writing training on reading and writing performance in preschool children. Adv Cogn Psychol. 2015;11(4):136-46. doi: 10.5709/acp-0178-7.	Non-adult
30	Bisio A, Pedullà L, Bonzano L, Ruggeri P, Brichetto G, Bove M. Evaluation of handwriting movement kinematics: From an ecological to a magnetic resonance environment. Front Hum Neurosci. 2016;10:488. doi: 10.3389/fnhum.2016.00488.	**Included**
31	Karimpoor M, Churchill NW, Tam F, Fischer CE, Schweizer TA, Graham SJ. Functional MRI of handwriting tasks: A study of healthy young adults interacting with a novel touch-sensitive tablet. Front Hum Neurosci. 2018;12:30. doi: 10.3389/fnhum.2018.00030.	**Included**
32	Hosni SM, Shedeed HA, Mabrouk MS, Tolba MF. EEG-EOG based virtual keyboard: Toward hybrid brain computer interface. Neuroinformatics. 2019;17(3):323-341. doi: 10.1007/s12021-018-9402-0.	Unfocused
33	Palmis S, Velay JL, Fabiani E, Nazarian B, Anton JL, Habib M, Kandel S, Longcamp M. The impact of spelling regularity on handwriting production: A coupled fMRI and kinematics study. Cortex. 2019;113:111-127. doi: 10.1016/j.cortex.2018.11.024. Epub 2018 Dec 10.	**Included**
34	Bartoň M, Fňašková M, Rektorová I, Mikl M, Mareček R, Rapcsak SZ, Rektor I. The role of the striatum in visuomotor integration during handwriting: an fMRI study. J Neural Transm (Vienna). 2020;127(3):331-337. doi: 10.1007/s00702-019-02131-8. Epub 2020 Jan 4.	**Included**

35	Askvik EO, van der Weel FRR, van der Meer ALH. The importance of cursive handwriting over typewriting for learning in the classroom: A high-density EEG study of 12-year-old children and young adults. Front Psychol. 2020;11:1810. doi: 10.3389/fpsyg.2020.01810.	**Included**
36	Lopez-de-Ipina K, Solé-Casals J, Sánchez-Méndez JI, Romero-Garcia R, Fernandez E, Requejo C, Poologaindran A, Faúndez-Zanuy M, Martí-Massó JF, Bergareche A, Suckling J. Analysis of fine motor skills in essential tremor: Combining neuroimaging and handwriting biomarkers for early management. Front Hum Neurosci. 2021;15:648573. doi: 10.3389/fnhum.2021.648573.	Unfocused
37	Watanabe H, Nakajima K, Takagi S, Mizuyama R, Saito M, Furusawa K, Nakatani K, Yokota Y, Kataoka H, Nakajima H, Naruse Y. Differences in mechanical parameters of keyboard switches modulate motor preparation: A wearable EEG study. Front Neuroergon. 2021;2:644449. doi: 10.3389/fnrgo.2021.644449.	No hw or ty
38	Boyraz RK, Kirpinar I, Yilmaz O, Özyurt O, Kiliçarslan T, Aralasmak A. A treatment-response comparison study of resting-state functional magnetic resonance imaging between standard treatment of SSRI and standard treatment of SSRI plus non-dominant hand-writing task in patients with major depressive disorder. Front Psychiatry. 2021;12:698954. doi: 10.3389/fpsyt.2021.698954.	**Included**
39	Zhang J, Kang L, Li J, Li Y, Bi H, Yang Y. Brain correlates of Chinese handwriting and their relation to reading development in children: An fMRI study. Brain Sci. 2022;12(12):1724. doi: 10.3390/brainsci12121724.	Non-adult
40	Van der Weel FRR, Van der Meer ALH. Handwriting but not typewriting leads to widespread brain connectivity: a high-density EEG study with implications for the classroom. Front Psychol. 2024;14:1219945. doi: 10.3389/fpsyg.2023.1219945.	**Included**
41	Yang X, Xiong X, Li X, Lian Q, Zhu J, Zhang J, Qi Y, Wang Y. Reconstructing multi-stroke characters from brain signals toward generalizable handwriting brain-computer interfaces. IEEE Trans Neural Syst Rehabil Eng. 2024;PP. doi: 10.1109/TNSRE.2024.3492191. Epub ahead of print 2024 Nov 6.	Unfocused
42	Schirripa Spagnolo G, Calabrese B, Ferrari G. Color separation to facilitate handwriting examination. Proceedings of the 5th International Symposium on Communications, Control and Signal Processing, ISCCSP 2012, Rome, Italy, 2-4 May 2012. IEEE Xplore: 21 June 2012: pp. 1-4, doi: 10.1109/ISCCSP.2012.6217832.	Unrelated
43	Berger CE, de Koeijer JA, Glas W, Madhuizen HT. Color separation in forensic image processing. J Forensic Sci. 2006;51(1):100-2. doi: 10.1111/j.1556-4029.2005.00020.x.	Unrelated
44	Danna J, Velay JL. On the auditory-proprioception substitution hypothesis: Movement sonification in two deafferented subjects learning to write new characters. Front Neurosci. 2017;11:137. doi: 10.3389/fnins.2017.00137.	Pathol prtcs
45	Longcamp M, Lagarrigue A, Nazarian B, Roth M, Anton JL, Alario FX, Velay JL. Functional specificity in the motor system: Evidence from coupled fMRI and kinematic recordings during letter and digit writing. Hum Brain Mapp. 2014;35(12):6077-87. doi: 10.1002/hbm.22606. Epub 2014 Aug 5.	**Included**
46	Planton S, Longcamp M, Péran P, Démonet JF, Jucla M. How specialized are writing-specific brain regions? An fMRI study of writing, drawing and oral spelling. Cortex. 2017;88:66-80. doi: 10.1016/j.cortex.2016.11.018. Epub 2016 Dec 18.	**Included**
47	Roux FE, Dufor O, Giussani C, Wamain Y, Draper L, Longcamp M, Démonet JF. The graphemic/motor frontal area Exner's area revisited. Ann Neurol. 2009;66(4):537-45. doi: 10.1002/ana.21804.	**Included**
48	Petrides M, Alivisatos B, Evans AC. Functional activation of the human ventrolateral frontal cortex during mnemonic retrieval of verbal information. Proc Natl Acad Sci U S A. 1995;92(13):5803-7. doi: 10.1073/pnas.92.13.5803.	**Included**
49	Seitz RJ, Canavan AG, Yágüez L, Herzog H, Tellmann L, Knorr U, Huang Y, Hömberg V. Representations of graphomotor trajectories in the human parietal cortex: evidence for controlled processing and automatic performance. Eur J Neurosci. 1997;9(2):378-89. doi: 10.1111/j.1460-9568.1997.tb01407.x.	**Included**
50	Tokunaga H, Nishikawa T, Ikejiri Y, Nakagawa Y, Yasuno F, Hashikawa K, Nishimura T, Sugita Y, Takeda M. Different neural substrates for Kanji and Kana writing: a PET study. Neuroreport. 1999;10(16):3315-9. doi: 10.1097/00001756-199911080-00012.	No hw or ty
51	Nakamura K, Honda M, Okada T, Hanakawa T, Toma K, Fukuyama H, Konishi J, Shibasaki H. Participation of the left posterior inferior temporal cortex in writing and mental recall of kanji orthography: A functional MRI study. Brain. 2000;123(Pt 5):954-67. doi: 10.1093/brain/123.5.954.	**Included**
52	Katanoda K, Yoshikawa K, Sugishita M. A functional MRI study on the neural substrates for writing. Hum Brain Mapp. 2001;13(1):34-42. doi: 10.1002/hbm.1023.	**Included**
53	Nakamura K, Honda M, Hirano S, Oga T, Sawamoto N, Hanakawa T, Inoue H, Ito J, Matsuda T, Fukuyama H, Shibasaki H. Modulation of the visual word retrieval system in writing: a functional MRI study on the Japanese orthographies. J Cogn Neurosci. 2002;14(1):104-15. doi: 10.1162/089892902317205366.	**Included**
54	Beeson PM, Rapcsak SZ, Plante E, Chargualaf J, Chung A, Johnson SC, Trouard TP. The neural substrates of writing: A functional magnetic resonance imaging study. Aphasiology. 2003;17(6-7):647-65. https://doi.org/10.1080/02687030344000067	**Included**
55	Omura K, Tsukamoto T, Kotani Y, Ohgami Y, Yoshikawa K. Neural correlates of phoneme-to-grapheme conversion. Neuroreport. 2004;15(6):949-53. doi: 10.1097/00001756-200404290-00004.	**Included**
56	Rektor I, Rektorová I, Mikl M, Brázdil M, Krupa P. An event-related fMRI study of self-paced alphabetically ordered writing of single letters. Exp Brain Res. 2006;173(1):79-85. doi: 10.1007/s00221-006-0369-y. Epub 2006 Feb 28. Erratum in: Exp Brain Res. 2006;173(1):193-4.	**Included**
57	Sugihara G, Kaminaga T, Sugishita M. Interindividual uniformity and variety of the "Writing center": a functional MRI study. Neuroimage. 2006;32(4):1837-49. doi: 10.1016/j.neuroimage.2006.05.035. Epub 2006 Jul 26.	No hw or ty
58	Harrington GS, Farias D, Davis CH, Buonocore MH. Comparison of the neural basis for imagined writing and drawing. Hum Brain Mapp. 2007;28(5):450-9. doi: 10.1002/hbm.20286.	**Included**
59	Brownsett SL, Wise RJ. The contribution of the parietal lobes to speaking and writing. Cereb Cortex. 2010;20(3):517-23. doi: 10.1093/cercor/bhp120. Epub 2009 Jun 16.	**Included**
60	Purcell JJ, Napoliello EM, Eden GF. A combined fMRI study of typed spelling and reading. Neuroimage. 2011;55(2):750-62. doi: 10.1016/j.neuroimage.2010.11.042. Epub 2010 Nov 23.	**Included**
61	Rapp B, Dufor O. The neurotopography of written word production: an fMRI investigation of the distribution of sensitivity to length and frequency. J Cogn Neurosci. 2011;23(12):4067-81. doi: 10.1162/jocn_a_00109. Epub 2011 Aug 3.	No hw or ty
62	Rapp B, Lipka K. The literate brain: the relationship between spelling and reading. J Cogn Neurosci. 2011;23(5):1180-97. doi: 10.1162/jocn.2010.21507. Epub 2010 Apr 30.	No hw or ty
63	Shah C, Erhard K, Ortheil HJ, Kaza E, Kessler C, Lotze M. Neural correlates of creative writing: an fMRI study. Hum Brain Mapp. 2013;34(5):1088-101. doi: 10.1002/hbm.21493. Epub 2011 Dec 8.	**Included**
64	Segal E, Petrides M. The anterior superior parietal lobule and its interactions with language and motor areas during writing. Eur J Neurosci. 2012;35(2):309-22. doi: 10.1111/j.1460-9568.2011.07937.x. Epub 2011 Dec 20.	**Included**

Included	30
Excluded	34
Unfocused	10
Unrelated	7
No actual writing or typing (No hw or ty)	5
Non-adult	3
No neuroimaging (No NI)	3
Reviews/meta-analyses (review)	2

Case reports/series (case)	2
Pathological participants (pathol prtcs)	1
Duplicates	1

Supplementary Table S2. Study locations.

Study	Institution-Location
Petrides et al., 1995	Montreal Neurological Institute, McGill University, Montréal, Québec, Canada
Seitz et al., 1997	Heinrich-Heine-Universität Düsseldorf, Nordrhein-Westfalen, Germany
Nakamura et al., 2000	Kyoto University Graduate School of Medicine-National Institute for Physiological Science, Kyoto, Japan
Katanoda et al., 2001	Institute of Medical Science, Faculty of Medicine, University of Tokyo, Japan
Matsuo et al., 2001	National Institute of Advanced Industrial Science and Technology, Osaka; Toyohashi Sozo College, Toyohashi; Kyoto University, Kyoto; Showa Women's University, Tokyo, Japan
Siebner et al., 2001	Departments of Neurology and Nuclear Medicine, Technische Universität München, Bayern, Germany
Siebner et al., 2002	Departments of Neurology and Nuclear Medicine, Technische Universität München, Bayern, Germany
Nakamura et al., 2002	Rakuwa-kai Otowa Hospital, Kyoto University, National Institute for Physiological Sciences, Okazaki, Japan
Beeson et al., 2003	University of Arizona and Southern Arizona Veteran's Affairs Medical Center, Tuscon, Arizona, USA
Omura et al., 2004	Institute of Medical Science, The University of Tokyo-Tokyo Metropolitan University- Tokyo Institute of Technology, Tokyo, Japan
Rektor et al., 2006	Department of Neurology, St. Anne's Hospital, Masaryk University, Brno, Czech Republic
Harrington et al., 2007	Departments of Radiology and Physical Medicine and Rehabilitation, University of California at Davis, Sacramento, California
Longcamp et al., 2008	Université Paul Sabatier, Toulouse, Université de la Méditerranée et Hôpital de La Timone, Marseille, France
Tremblay et al., 2008	Faculty of Medicine, School of Communication Sciences and Disorders, and Centre for Research on Language, Mind and Brain, McGill University, Montréal, Québec, Canada
Roux et al., 2009	INSERM, Imagerie cérébrale et handicaps neurologiques, University of Toulouse; Pole Neurosciences, Centres Hospitalo-Universitaires, and LAPMA, Paul Sabatier University, Toulouse, France
Barton et al., 2010	University of British Columbia and Vancouver General Hospital, Vancouver, British Columbia, Canada
Brownsett & Wise, 2010	Division of Neuroscience and Mental Health and Medical Research Council Clinical Sciences Centre, Imperial College, Hammersmith Campus, London, UK
Purcell et al., 2011	Center for the Study of Learning & Center for the Study of Visual Language and Visual Learning, Georgetown University, Washington, DC, USA
Segal & Petrides, 2012	Cognitive Neuroscience Unit, Montreal Neurological Institute, McGill University, Montréal, Québec, Canada
Shah et al., 2013	Departments of Neurology and Institute of Diagnostic Radiology and Neuroradiology, Universität Greifswald, Mecklenburg-Vorpommern; Institute for Creative Writing and Cultural Journalism, Universität Hildesheim, Niedersachsen, Germany
Longcamp et al., 2014	CNRS, Aix Marseille Université, Marseille, France
Higashiyama et al., 2015	Department of Neurology and Stroke Medicine, Yokohama City University Graduate School of Medicine, Yokohama; Department of Neurology, International University of Health and Welfare, Mita Hospital, Tokyo; Center for Advanced Research for Logic and Sensibility, Keio University, Tokyo, Japan
Bisio et al., 2016	Dipartimento di Medicina Sperimentale; Dipartimento di Neuroscienze, riabilitazione, oftalmologia, genetica e scienze materno-infantili, Università di Genova, Fondazione Italiana Sclerosi Multipla, Genova, Liguria, Italy
Planton et al., 2017	ToNIC-Toulouse NeuroImaging Center-URI Octogone-Lordat, Université de Toulouse, Inserm, France
Karimpour et al., 2018	Department of Medical Biophysics, Sunnybrook Research Institute-Departments of Neurosurgery and Psychiatry, University of Toronto, Toronto, Ontario, Canada
Palmis et al., 2019	Laboratoire de Neurosciences Cognitives-Institut de Neurosciences de la Timone, CNRS–Université Aix-Marseille, Marseille, France
Bartoň et al., 2020	CEITEC-Central European Institute of Technology, Multimodal and Functional Neuroimaging and Applied Neurosciences Research Groups, Masarykova univerzita, Brno, Czech Republic
Askvik et al., 2020	Institutt for psykologi, NuLab-Developmental Neuroscience Department, Norges teknisk-naturvitenskapelige universitet, Trondheim, Norway
Boyraz et al., 2021	Bezmiâlem Vakif Üniversitesi, Psikiyatri Anabilim Dalı-Radyoloji Anabilim Dalı, Tıp Fakültesi, Fatih-Bogaziçi Üniversitesi, Bogaziçi, Mühendislik Fakültesi, Beşiktaş, İstanbul, Türkiye
Van der Weel and Van der Meer, 2024	Institutt for psykologi, NuLab-Developmental Neuroscience Department, Norges teknisk-naturvitenskapelige universitet, Trondheim, Norway
